# Development and Characterization of Electrodes Coated with Plasma-Synthesized Polypyrrole Doped with Iodine, Implanted in the Rat Brain Subthalamic Nucleus

**DOI:** 10.3390/polym16060823

**Published:** 2024-03-15

**Authors:** Daniel Ruiz-Diaz, Joaquín Manjarrez-Marmolejo, Araceli Diaz-Ruiz, Camilo Ríos, María G. Olayo, Roberto Olayo, Guillermo J. Cruz, Hermelinda Salgado-Ceballos, Marisela Mendez-Aramenta, Juan Morales-Corona

**Affiliations:** 1 Biomedical Engineering Grad School, Universidad Autónoma Metropolitana Iztapalapa, Mexico City 09340, Mexico; danielruiz2425@gmail.com; 2 Laboratory of Physiology of the Reticular Formation, National Institute of Neurology and Neurosurgery Manuel Velasco Suárez, Mexico City 14269, Mexico; 3 Department of Neurochemistry, National Institute of Neurology and Neurosurgery Manuel Velasco Suárez, Mexico City 14269, Mexico; 4 Research Directorate, National Institute of Rehabilitation Luis Guillermo Ibarra Ibarra, Mexico City 14389, Mexico; 5 Department of Physics, National Institute of Nuclear Research, Ocoyoacac 52750, Mexico; 6 Department of Physics, Universidad Autónoma Metropolitana Iztapalapa, Mexico City 09340, Mexico; 7 Medical Research Unit in Neurological Diseases, Specialty Hospital Centro Médico Nacional Siglo XXI, Mexican Institute of Social Security, Mexico City 06703, Mexico

**Keywords:** implanted electrodes, biomaterials, plasma synthesis, polypyrrole doped with iodine, Parkinson’s disease, plasma synthesis, electrographic recordings

## Abstract

Biological treatments involve the application of metallic material coatings to enhance biocompatibility and properties. In invasive therapies, metallic electrodes are utilized, which are implanted in patients. One of these invasive therapeutic procedures is deep brain stimulation (DBS), an effective therapy for addressing the motor disorders observed in patients with Parkinson’s disease (PD). This therapy involves the implantation of electrodes (IEs) into the subthalamic nucleus (STN). However, there is still a need for the optimization of these electrodes. Plasma-synthesized polypyrrole doped with iodine (PPPy/I) has been reported as a biocompatible and anti-inflammatory biomaterial that promotes nervous system regeneration. Given this information, the objective of the present study was to develop and characterize a PPPy/I-coated electrode for implantation into the STN. The characterization results indicate a uniform coating along the electrode, and physical–chemical characterization studies were conducted on the polymer. Subsequently, the IEs, both coated and uncoated with PPPy/I, were implanted into the STN of male rats of the Wistar strain to conduct an electrographic recording (EG-R) study. The results demonstrate that the IE coated with PPPy/I exhibited superior power and frequency signals over time compared to the uncoated IE (*p* < 0.05). Based on these findings, we conclude that an IE coated with PPPy/I has optimized functional performance, with enhanced integrity and superior signal quality compared to an uncoated IE. Therefore, we consider this a promising technological development that could significantly improve functional outcomes for patients undergoing invasive brain therapies.

## 1. Introduction

Electrodes play a crucial role in biomedical research, allowing for electrical stimulation and the recording of cellular and tissue responses [1]. Over time, extensive investigations have been conducted to understand the body’s reaction to various materials, starting with natural materials and progressing to synthetic materials or their combinations and alloys [2]. Generally, studies in this field can be categorized into three divisions: toxic, reactive, and non-reactive materials [3,4]. The use of these biomaterials has become imperative in various invasive therapies, prompting a dedicated field of study aimed at improving their interaction with bodily tissues.

One such biocompatible material is polypyrrole (PPy), composed of heterocyclic molecules with five members, including four carbons and one nitrogen, featuring conjugated double bonds [5]. Due to their broadly tailorable properties, PPy coatings have a broad range of different biomedical application areas, such as tissue engineering scaffolds, implant coatings, drug delivery devices, and recording and stimulation applications [6]. Electrically conductive polymers, such as PPy, have undergone extensive research for their applications as active electrode materials and supporting additives across various domains, including electronics, batteries, electrodes, and biomaterials [7].

Polypyrrole stands out as a novel biocompatible, biostable, and semiconductor material, with its biological applications tested both in vitro and in vivo. By leveraging its electrical and biological properties, PPy can be employed in the field of bioengineering to create novel technologies, including cellular functions like migration, adhesion, DNA synthesis, and protein secretion, ultimately contributing to tissue repair and other biological processes [8].

To enhance metal corrosion resistance and increase biocompatibility, PPy is frequently used as a coating on metals through various techniques [9,10]. Additionally, PPy is synthesized with a dopant to enhance its biological interaction with tissues or directly with the metal. Many studies on metal coatings with PPy involve electrochemical synthesis [11,12], often requiring the use of specific precursors to facilitate synthesis, including initiators, propagators, or terminators. Notably, PPy’s conductivity is significantly influenced by the presence of other elements or compounds within its structure, such as chemical dopants, ion implantation, copolymerization, or metal complexes [13].

The synthesis of this polymer by plasma involves the use of the monomer, the dopant, and the electrons released in the plasma, resulting in a material free of impurities. Cross-linked polypyrroles replace hydrogen with another pyrrole ring, allowing for the construction of three-dimensional surfaces or networks of pyrrole rings. This effect cannot be achieved with linear polypyrroles due to the lack of multidirectional growth seen in their cross-linked counterparts. The structural resemblance of highly cross-linked polypyrrole [14,15] makes it a valuable polymer for coating surfaces such as implantable electrodes (IEs).

Polypyrrole has not only been used as a coating but also extensively studied in a wide variety of polymeric scaffolds for its application in implantable and temporary devices in tissue engineering. Biocompatible scaffolds with open porous structures and adequate mechanical resistance provide an optimal microenvironment for tissue proliferation, cell migration, differentiation, and the guidance of cell growth in the recipient tissue [16].

Plasma-synthesized iodine-doped polypyrrole (PPPy/I) has been demonstrated to be a highly cross-linked and branched material, insoluble, and thermally stable. Its electrical properties improve with iodine doping and increasing relative humidity [17]. Our research group presented experimental evidence involving rats and rhesus monkeys regarding the biocompatibility of PPPy/I. The results indicate that the implants were integrated into the nervous tissue of the previously injured spinal cord with lower inflammatory and gliotic responses compared to the control group (without implants). There was no evidence of implant rejection, and PPPy/I also prevented secondary tissue destruction [18].

PPPy/I has found application as a surface coating for stents to mitigate foreign body tissue reactions. Static and dynamic models were employed to assess the binding of stents and PPPy/I when fully immersed in phosphate-buffered saline (PBS) for 2 months [19].

This work entails the development of electrodes coated with PPPy/I for brain implantation in rats, with the aim of enhancing biocompatibility and recording electrical activity.

## 2. Materials and Methods

### 2.1. Design of the IE

**Figure 1:**
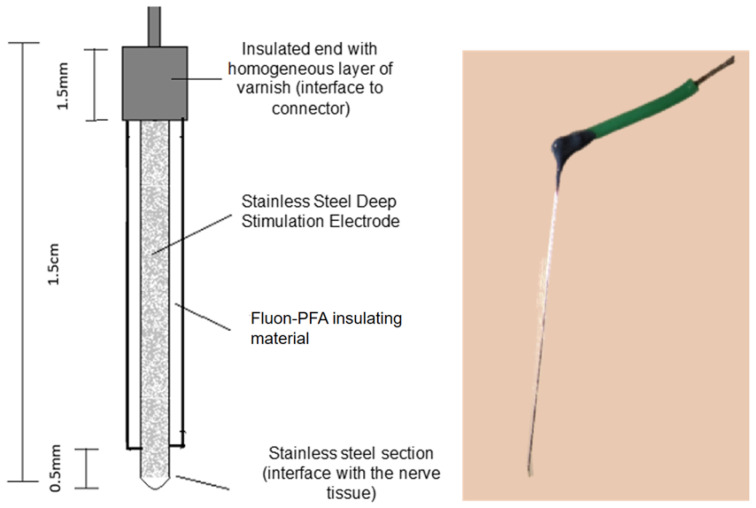
Design of the implanted electrode, total diameter of 139.7 μm, stainless steel of 76.2 μm, and 63.5 μm thick Fluon-PFA. On right side, a representative real electrode.

For the design and fabrication of deep stimulation electrodes, we utilized a stainless steel (SS) microwire (A-M SYSTEM STM)^®^ with a diameter of 76.2 μm and a Fluon^®^- Perfluoroalkoxy (PFA) insulating coating of 63.5 μm in thickness. The total length of the microelectrode was 1.5 cm, with 0.5 mm of the insulating material removed from one end, leaving only the metal in contact with the nerve tissue to facilitate electrographic recording (EG-R). At the opposite end (interface), 1.5 mm of the insulating PFA was removed, and the microwire was then securely attached to a copper wire using tin solder. Finally, the assembly was coated with a uniform layer of varnish as an additional insulating material (Figure 1).

### 2.2. Abrasion and Synthesis of Polypyrrole Doped with Iodine Coating

A plasma reactor was employed for coating the electrodes (IEs). The reactor featured stainless steel flanges at both ends, each equipped with three access ports. Pyrrole (Sigma-Aldrich, St. Louis, MO, USA, 99%) and iodine (Sigma-Aldrich, 99.8%) were introduced into the reactor through these ports for the synthesis of PPPy/I, or the ports were sealed during the abrasion process. In the other ports, a vacuum mechanical pump (Edwards) and cold traps with liquid nitrogen were connected to the reactor to lower the pressure. Metallic electrodes were inserted in the center of the flanges to create a uniform electric field within the reactor.

The IEs were positioned in the center of the tubular glass reactor, with a 5 cm gap between the electrodes responsible for the glow discharge. These electrodes were connected to a Dressler Cesar-1500 generator operating at a 13.56 MHz RF frequency. Subsequently, the IEs underwent an abrasion process within the reactor to sensitize their surfaces and generate micropores, thereby enhancing the adhesion of the PPPy/I coating.

The parameters used in the plasma reactor were as follows: a frequency of 13.56 MHz, an average pressure of 9 × 10^−1^ Torr, and a power of 100 W. The power was gradually increased over five minutes, starting at 20 W for the first minute and reaching 100 W by the fifth minute, to avoid causing inadequate abrasion that might damage or burn the electrode materials. The power was similarly decreased over the last 5 min, resulting in a total abrasion time of 45 min. In the abrasion process, the reactor environment was the residual atmosphere; no other gas was used for this process.

Following the abrasion process, the synthesis of the polypyrrole doped with iodine coating was initiated using radiofrequency glow discharges. Plasma was generated as electric glow discharges at an approximate pressure of 5 × 10^−1^ Torr and a power of 20 W. The reactions commenced in the gas phase and concluded in the solid phase as thin films adhered to the IE surface. Polymerization occurred over a 20 min duration, with the first 6 min involving pyrrole only, followed by a 4 min mix with iodine, repeating this process twice to achieve a total polymerization time of 20 min. The chamber was flooded with vapors of pyrrole and iodine during the discharges. No carrier gas was used to dilute or contaminate the flow of the reagents.

### 2.3. Polymer Characterization

#### 2.3.1. Infrared Spectroscopy by Attenuated Total Reflectance (FTIR-ATR)

The structure of the polymer coating was analyzed using infrared spectroscopy, employing a Miracle Single Reflection Horizontal ATR Accessory (PIKE) with a diamond/ZnSe crystal and 32 scans. Attenuated total reflectance (ATR) was utilized to examine the surface of the polymer, where interactions between the polymer and cells occur.

#### 2.3.2. X-ray Photoelectron Spectroscopy (XPS)

XPS spectroscopy was conducted using a Thermo Scientific, Waltham, MA, USA, TM K-Alpha spectrometer equipped with a monochromatic Al source (1486.6 eV) operated in survey mode for XPS analyses. The samples were deposited on Al tape, which was located and grounded onto the spectrometer stage. The pass energy in the survey mode was 200 eV. The electrostatic charges were partially neutralized with an air ion flood beam on the sample surface. The analysis area had an approximate diameter of 0.05 mm. Only the survey data with the elemental content are presented in the manuscript.

#### 2.3.3. Raman Spectroscopy

Raman spectroscopy was performed using a WiTec Raman System Alpha 300RA confocal microscope. The electrodes were exposed to a 532 nm laser light at a power of 75 mW. The electrodes were placed onto the Raman system stage without any additional treatment.

#### 2.3.4. Electrochemical Characterization

**Figure 2:**
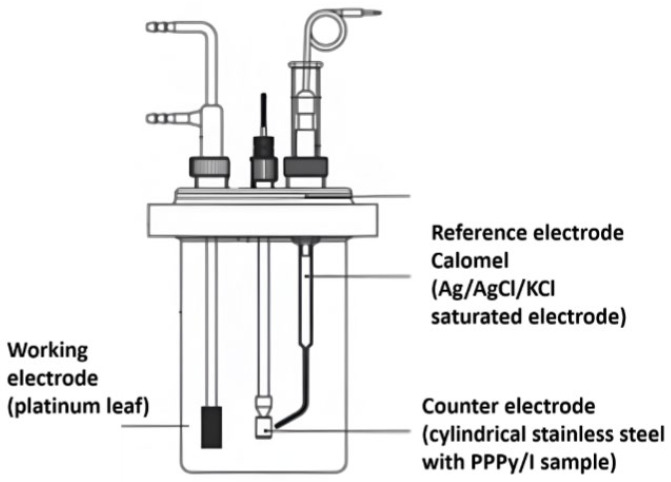
Electrochemical cell used to perform impedance and cyclic voltammetry studies in IE.

An electrochemical analysis was conducted using a conventional three-electrode cell with 1M potassium chloride as the electrolyte. A platinum leaf (3 cm × 2 cm) served as the working electrode, while an Ag/AgCl/KCl saturated electrode was used as the reference electrode (3M). The counter electrode was a stainless steel bar with a superficial coating of PPPy/I (Figure 2). Prior to analysis, dissolved oxygen was eliminated by purging the system with nitrogen.

#### 2.3.5. Scanning Electron Microscopy

A morphological analysis was conducted using a high-resolution scanning electron microscope (HRSEM JEOL JSM-7600F). The samples underwent gold sputtering before the examination (Denton Vaccum Desk II); the deposit was made at a pressure of 50 millitorr (mT) and a current of 25–30 mA, the time was 120 s, and the current of the images was 85 μA according to the tables in the microscope manual. The power used in the microscope is shown in the micrographs.

### 2.4. Biological Characterization

#### 2.4.1. Animals

All experiments were conducted in accordance with the technical specifications outlined in the Official Mexican Norm NOM 0062-ZOO-1999. The experimental procedures received approval from the Bioethical Committee of the Instituto Nacional de Neurología y Neurocirugía Manuel Velasco Suárez (Registration number: 91/13) in compliance with institutional guidelines and the Care and Use of Laboratory Animals regulations of the National Institutes of Health (USA).

Fourteen healthy male Wistar rats weighing between 270 and 300 g were included in the study. Throughout the protocol, the rats were housed under a 12 h light–dark cycle at a room temperature of 22 °C and provided ad libitum access to food and water.

#### 2.4.2. Implanting of Electrodes in the Subthalamic Nucleus

Eleven male Wistar rats were anesthetized with intraperitoneal ketamine and xylazine at doses of 80 mg/kg and 10 mg/kg, respectively, and their heads were securely fixed within a stereotactic frame. The Bregma reference point was identified, and in one group, a bipolar stainless steel PPPy/I-coated IE was unilaterally implanted into the subthalamic nucleus (STN) using the following coordinates: Anteroposterior (AP): −3.6 mm, Lateral (L): 2.5 mm, and Ventral (V): 7.7 mm, in accordance with Paxinos and Watson 2004.

In another group of rats, we implanted bipolar stainless steel electrodes with identical characteristics but without the PPPy/I coating. Additionally, a monopolar stainless steel electrode was positioned in the frontal bone as a ground electrode, and stainless steel anchor screws were secured to the skull. The entire assembly was affixed to the skull using dental cement.

Additionally, all the animals were implanted with a 25-gauge guide cannula of 18 mm in size; this cannula was located 4 mm above the target nucleus, in the striatal region, with coordinates set at AP: 0.5 mm, L: 3.0 mm, and V: 5.5 mm [20]. A stylet was used to seal the cannula for future use.

After the surgical procedures, the rats were granted a 1-week recovery period in their home cages, with access to food and water ad libitum, under a 12:12 h light–dark cycle with lights on at 07:00 h. Throughout the three days post-surgery, enrofloxacin (10 mg/kg orally) and meloxicam (1 mg/kg intraperitoneally) were administered as an antibiotic and analgesic, respectively.

A week after the surgical procedures, all animals received a microinjection of 10 μg/8 μL of 1-methyl-4-phenylpyridinium (MPP+), dissolved in sterile saline [21], with a 30-gauge stainless steel cannula of 22 mm in size attached to a Hamilton syringe by flexible tubing [22]. Please note that the complete results obtained from the MPP+ lesion will be presented in a separate study.

#### 2.4.3. Electrographic Recordings (EG-R)

The rats were connected to a model BE light amplifier (EBNeuro^®^, Firenze, Italy) using flexible cables, allowing them free movement within the acrylic boxes. Video-electrographic recordings (EG-Rs) were conducted using Galileo NT software (version 3.00/00, EBNeuro^®^, Firenze, Italy). These EG-R sessions were conducted weekly, with each session lasting 20 min over 10 weeks. All video recordings were stored on a hard disk for subsequent analysis of both power and frequency signals.

The video-electrographic signals underwent filtering, with a low-pass filter set at 0.3 Hz and a high-pass filter at 70 Hz to ensure signal clarity. Additionally, external noise interference was mitigated using a 60 Hz notch filter. During the rats’ wakefulness, an analysis was performed on 10-second epochs of video-EG-R data. Subsequently, an automated analysis based on the fast Fourier transform method was applied to estimate the total spectral power (μV2) of the video-EG-R signal.

### 2.5. Statistical Analysis

The statistical analysis of the acquired data involved assessing normal distribution using the Kolmogorov–Smirnov test and checking for homogeneity of variances through Levene’s test. Differences in total spectral power, frequency, and behavioral assessments were evaluated using a repeated-measures ANOVA test. All analyses were conducted using SPSS 22.0 software (Chicago, IL, USA). Statistical significance was established at *p* < 0.05.

## 3. Results

### 3.1. Characterization of the Coating with Plasma-Synthesized Polypyrrole Doped with Iodine

**Figure 3:**
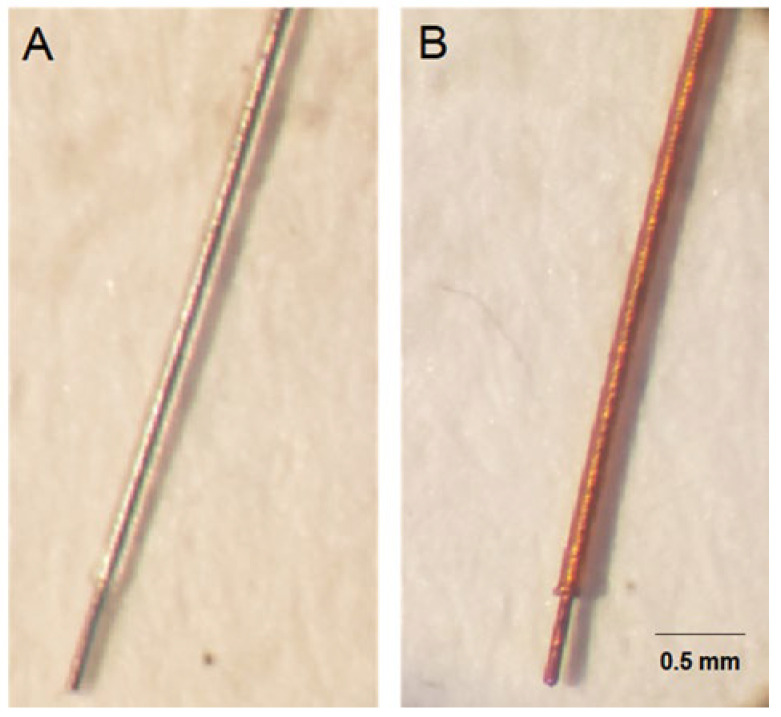
(**A**) The stainless steel electrode (SS-PFA) without PPPy/I coating, and (**B**) the same electrode coated with PPPy/I (SS-PFA-PPPy/I).

Figure 3 displays two images: Figure 3A shows the stainless steel wire (SS-PFA) devoid of the PPPy/I coating, and Figure 3B shows the same wire coated with PPPy/I (SS-PFA-PPPyI), exhibiting the distinctive amber hue associated with polypyrrole. A strategic design choice for this electrode was to leave a 0.5 cm tip uncoated with Teflon, enhancing the efficient flow of the electric current when it was inserted into the STN of the rat.

### 3.2. Structural Analysis by Infrared Spectroscopy (FTIR-ATR)

**Figure 4:**
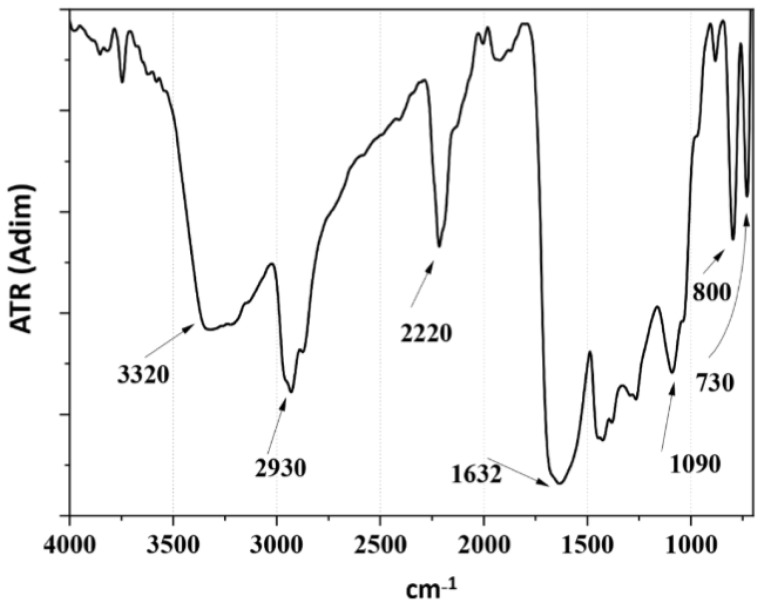
Infrared spectroscopy (FTIR-ATR) spectrum of the PPPy/I coating.

Figure 4 presents the FTIR-ATR spectra of PPPy/I, revealing the characteristic broad and intricate bands typical of materials synthesized via plasma processes. Notably, at 3351 cm^−1^, one can observe the symmetrical vibration in the stretching mode of primary and secondary amines. In proximity to this region, at 2958 cm, vibrations associated with aliphatic carbons in symmetrical and asymmetrical stretching modes are evident. At 2218 cm^−1^, we can discern the classical vibration of nitrile and alkyl groups (C≡N, C≡C), indicating the fragmentation of the pyrrole ring during plasma synthesis. The signal at 1681 cm^−1^ suggests vibrations in the plane of C=C or C=N.

Furthermore, the band at 739 cm^−1^ corresponds to the symmetric vibration of the polymer skeleton and signifies the presence of iodine, characteristic of the halogen family.

### 3.3. X-ray Photoelectron Spectroscopy (XPS)

**Figure 5:**
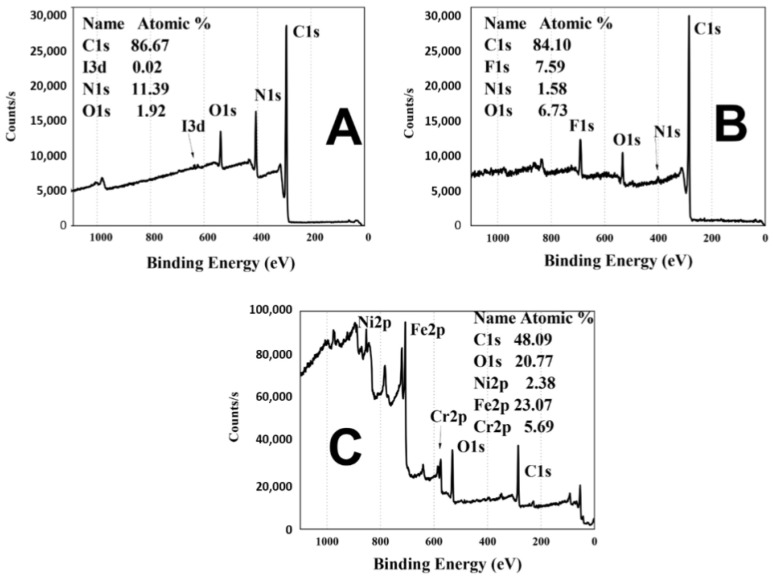
(**A**) The XPS spectrum of the surface coating of the electrode made by PPPy/I, and the C, N and O elements characteristic of PPPy/I deposited by plasma. (**B**) The XPS of the PPPy/I coating and the PFA layer is shown due to the appearance of F. (**C**) The XPS spectrum of the active tip of the electrical stimulation electrode; the presence of F does not show that, in this section, the PFA coating was removed from the stainless steel wire, showing the classical elements of SS and the PPPy/I coating that covers the stainless steel.

In Figure 5, the XPS analysis of various electrodes is presented. In Figure 5A, we observe the spectra of the PPPy/I film, which consists of 86.67% carbon (C), a mere 0.02% iodine (I), 11.39% nitrogen (N), and 1.92% oxygen (O). Notably, it should be emphasized that the pyrrole monomer lacks oxygen in its elemental composition. At the conclusion of the polymerization reaction, unreacted radicals interact with the atmosphere, leading to the incorporation of oxygen into the surface layer of the polymer.

Figure 5B showcases the surface of the PFA-coated wire with the PPPy/I layer, containing 84.1% carbon (C), 1.58% nitrogen (N), and 6.73% oxygen (O) and exhibiting the presence of fluorine (F) at 7.59%. This fluorine originates from the PFA layer on the stainless steel, elucidating the increased carbon and fluorine content and the reduced nitrogen levels. The signal for iodine in the studied material is no longer discernible.

Figure 5C provides the XPS spectrum of the active contact of the microelectrode. This spectrum reveals the presence of nickel (Ni), iron (Fe), and chromium (Cr) at concentrations of 2.38%, 23.07%, and 5.69%, respectively. Additionally, oxygen (O) accounts for 20.77% of the composition, while carbon (C) constitutes 48.09%. In this spectrum, the signals for nitrogen (N) and iodine (I) are scarcely noticeable, potentially attributed to noise due to their minimal presence compared to the other elements. Spectrum (A) indicates that the conditions of PPPy/I synthesis effectively cover the Teflon insulation layer, the stainless steel, and even the 0.5 cm tip where the PFA cover was removed.

### 3.4. Raman Spectroscopy

**Figure 6:**
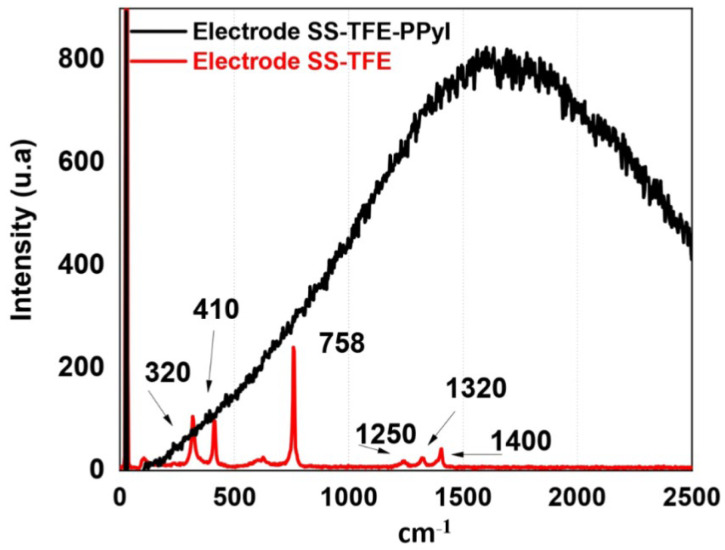
Raman spectrum of electrodes: red line, without PPPy/I coating; black line, with PPPy/I coating.

Figure 6 depicts the Raman spectrum of the SS-PTFE (red line) and SS-PTFE-PPPy/I (black line) electrodes. In the SS-PTFE spectrum, lines at 758 and 1400 cm^−1^ are evident, attributed to the symmetric stretching vibration of CF2 and C–C, respectively. Overtone and combination vibrational modes are closely situated at 1250 and 1320 cm^−1^. The signals at 320 and 410 cm^−1^ can be ascribed to the stainless steel structure.

In the same figure, it is apparent that PPPy/I displays a broad and intricate band characteristic of the fluorescence associated with the plasma-synthesized PPy/I coating. Within this complex signal, the contributions from PFA and stainless steel are not discernible [23].

### 3.5. Electrochemical Characterization

**Figure 7:**
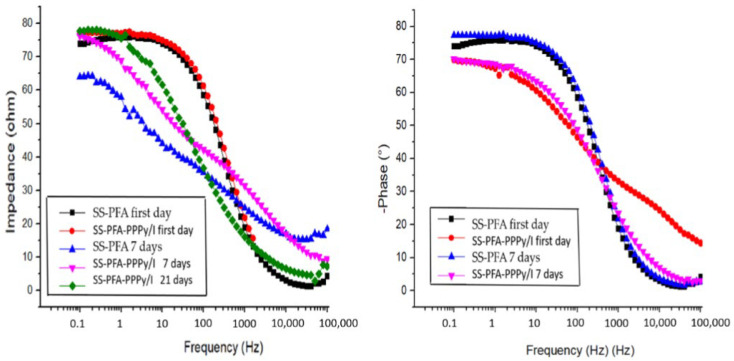
Impedance vs. frequency and phase angle vs. logarithm of the frequency of the SS-PFA and SS-PFA-PPPy/I.

Figure 7 presents Bode plots illustrating impedance versus frequency and the logarithm of the phase angle versus frequency for the SS-PTFE and SS-PTFE-PPPy/I electrodes. These spectra were generated by immersing the electrodes in a Krebs–Ringer solution and recording data on days 1, 7, and 21 of immersion in the K-B solution.

As observed, the SS-PFA electrode remains unaltered when submerged in the Krebs–Ringer solution, indicating the stability of its passive layer. In contrast, the SS-PFA-PPPy/I electrode exhibits an increasing impedance over time, indicative of the gradual stabilization of its layer. At frequencies exceeding 200 Hz, it demonstrates a more robust protective layer compared to SS-PFA. This enhanced stability translates to improved performance in electrocorticography (EG-R) experiments with rats.

**Figure 8:**
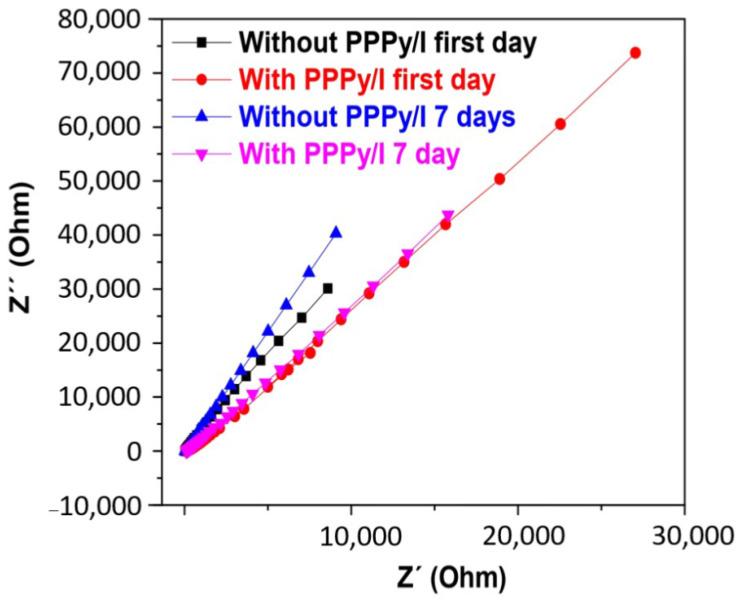
Nyquist diagram of SS-PFA-PPPy/I and SS-PFA.

The Nyquist diagram presented in Figure 8 illustrates that the resistance of the SS-PFA-PPPy/I electrode is notably higher when compared to that of SS-PFA. This observation suggests that the PPPy/I layer enhances its stability and exhibits reduced solubility in the working solution. Notably, all surfaces display a high phase angle, signifying that the passive layers effectively confer protective properties to the metal electrodes. Furthermore, it becomes evident that, beyond 200 Hz, the PPPy/I layer imparts greater stability to the protective layer.

### 3.6. Scanning Electron Microscopy

**Figure 9:**
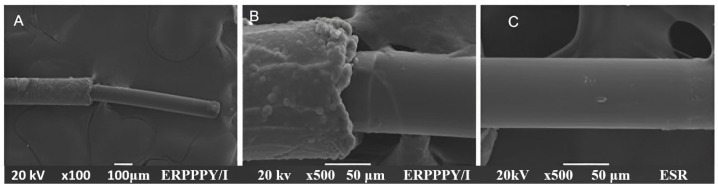
SEM of the stainless steel PFA plasma-synthesized iodine-doped polypyrrole electrode (**A**) panoramic view of the electrode, (**B**) closer view of the interface between the PPPy/I coating and the stainless steel, (**C**) stainless steel wire covered by PPPy/I.

Regarding the coverage of the stainless steel PFA-coated wires, the SEM analysis confirms the complete coverage of both the metal and Teflon layers. Figure 9 presents the SEM images of a single SS-PFA-PPPy/I wire. In Figure 9A, a panoramic view of the electrode reveals a 0.5 cm tip thinly coated with PPPy/I, while the bulkier section corresponds to the metal coated with PFA and PPPy/I. Figure 9B offers a closer view of the interface between the PPPy/I coating and the stainless steel. Notably, the PFA cover exhibits a rough texture with spherical particles, which appear to coalesce and form the PPPy/I cover. Upon zooming in closer to this area, the PFA coating on the metal becomes discernible. Figure 9C provides an image of the stainless steel wire covered by PPPy/I, showcasing a generally smooth coating with scattered spherical particles on the surface. The XPS analysis confirms the complete coverage of this zone with the PPPy/I film.

### 3.7. Video-Electrographic Recordings (vEG-Rs) of the Subthalamic Nucleus

**Figure 10:**
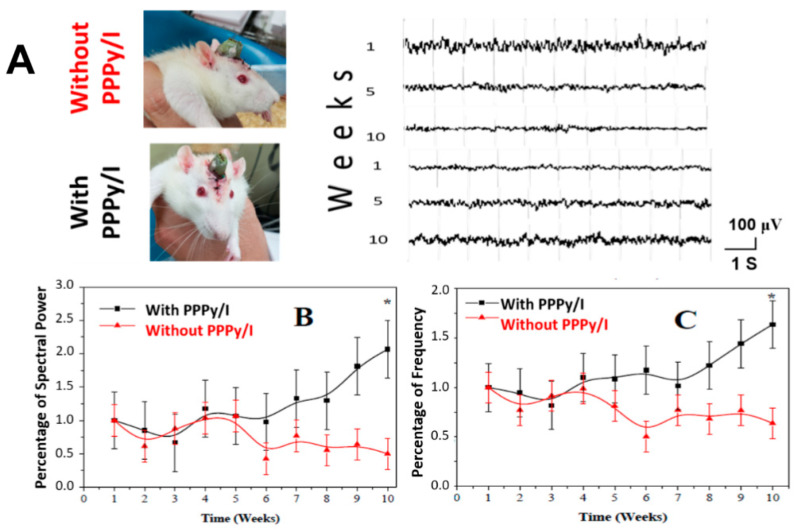
(**A**) Representative STN EG-Rs for 10 weeks, without PPPy/I and with PPPy/I. (**B**) The results of the electrical activity, measured as the total spectral power through the fast Fourier transform method to estimate the total spectral power (μV^2^) of the EG-R signal to examine the effect of the two different implanted electrodes (IEs); the values are expressed as a percentage for the different evaluation times. (**C**) Effect of IE on EG-R frequency (Hz) expressed as a percentage. The values of (**B**,**C**) are expressed as a percent of the basal mean (first week). The results are shown as average ± SEM and were analyzed using a repeated-measures ANOVA test. * *p* < 0.05.

Figure 10A displays representative EG-R data obtained in 10-second epochs within the subthalamic nucleus (STN) at 1, 5, and 10 weeks after electrode implantation, both with PPPy/I and without PPPy/I coatings. Figure 10B presents the results of electrical activity, quantified as the total spectral power using fast Fourier transform during these 10-second intervals. The values are expressed as a percentage of the baseline mean (first week) ± SEM. Significant differences were observed between the two groups (*p* < 0.05, repeated-measures one-way ANOVA test). Figure 10C presents the frequency results, expressed as a percentage of the baseline mean (first week) ± SEM. Statistically significant differences were observed between the groups (*p* < 0.05, repeated-measures one-way ANOVA test).

## 4. Discussion

The use of intracranial electrodes (IEs) is highly relevant for the treatment of various diseases, including obsessive–compulsive disorders [24], epilepsy [25], chronic pain [26], and PD [27]. The study of intracranial electrodes involves the development of electrodes with surfaces that interact effectively with tissue, are biocompatible, and possess suitable electrical properties for stimulation [28]. In this context, their use is essential for obtaining electrical records. In the case of PPy, the presence of bipolarons, polymeric structures comprising cations and anionic counterparts, allows the material to deliver the electric current more efficiently to cells [29].

The conductive properties of PPy have also been used to enhance cell proliferation and differentiation [30,31]. In neurons specifically, electrical stimulation using polypyrrole surfaces induced neurite growth and alignment in vitro, showing the possible effects of PPy-coated electrodes on neural plasticity [32], which may perhaps restore lost neuronal functions. In various studies, as reviewed recently [33], PPy has been employed as a coating material, synthesized either chemically or electrochemically, with the incorporation of various dopants, and with electrical stimulation.

In this article, we present the manufacturing of intracranial electrodes (IEs) utilizing PPy synthesized through plasma treatment, with the addition of iodine as a dopant to enhance its electrical properties. We also conduct a comprehensive physical characterization of the surface and an electrochemical analysis of the IEs. Additionally, we demonstrate an in vivo recording of the subthalamic nucleus (STN) electrical activity over 10 weeks; note that, at 10 weeks post-implantation of the electrodes with PPPy/I, the electrographic recording is better, indicating that the currents are efficiently transported back and forth between neurons through the PPPy/I interface of the electrode.

PPPy/I has found applications in spinal cord injuries [18], stents [19], and other areas. However, it has not been explored for more specific electrical applications, such as electrographic recordings. In this study, we present the results of a comparison between PPPy/I-coated electrodes with an approximately 2 μm coating thickness and uncoated stainless steel electrodes. This thickness was achieved with the deposition rate described by Cruz et al [14]. We conducted a thorough physical characterization to assess coating uniformity and compared changes through electrochemical characterization. We also conducted a 10-week follow-up study on the PPPy/I-coated intracranial electrodes (IEs), assessing electrographic recordings (EGRs) in terms of power and frequency values under identical conditions in male Wistar rats.

The decision to use PPPy/I was based on its demonstrated biocompatible properties in spinal cord injury applications and the necessity for a corrosion-resistant coating that protects tissue from metal-induced chemical changes. A prior abrasion process improved polymer adhesion to the electrode, ensuring a uniform coating that remained intact throughout the in vivo study. It is worth noting that the thickness of the coating significantly impacts its adhesion and electrical properties; thicker coatings are more prone to detachment and can become insulating. As a result, prior studies typically treat PPPy/I coatings as thin films [34,35].

Our spectroscopic analyses allowed us to observe and compare the different groups and elements within the PPPy/I, stainless steel, and insulating Teflon layer. We also noted a fluorescence effect in Raman microscopy attributed to PPy/I synthesis through plasma, a phenomenon not reported in other works using the electrochemical synthesis of polypyrrole. This study spanned 10 weeks, as it is intended for potential use in PD therapy, a chronic neurodegenerative condition that necessitates long-term treatment. We observed improvements in the subthalamic nucleus (STN).

It has been shown that a PPy coating benefits neural cell growth when it is electrically stimulated [36], but this has been tested in in vitro work, and important factors that may reduce electrical conductivity in vivo have not been taken into account. In the case of PPPy/I, it has been proven that neuronal tissue grows without electrical stimulation [18]. The use of an electrode with PPPy/I is in fact an electrical stimulus for the polymer and improves the interaction with neurons. The benefit may also be associated with the reaction of the brain tissue, particularly the foreign body rejection process, such as the inflammatory process, the action of macrophages, and the generation of microglia, which causes neurons to move away from the foreign body [37,38,39]. The improvement observed in EEGR data acquisition may be associated with a decrease in these processes. According to the results obtained, the electrodes with the PPPy/I coating could have a better interaction with neurons or a better biocompatibility process compared to those without the coating. We attribute the improvement to better polymer–tissue interactions, a potential reduction in glial scar thickness at the injury site, or increased PPPy/I conductivity under moist conditions [17], all factors contributing to the better acquisition of EGRs. However, conduction may be performed at both ends of the electrode, precluding a more efficient delivery of change to neurons in the case of electrode stimulation, employed, for example, to control Parkinson´s disease patients.

## 5. Conclusions

In this article, we successfully developed PPPy/I coatings for intracranial electrodes (IEs) aimed at enhancing the acquisition of electrical signals, particularly those originating from the STN. Our study demonstrated that, despite its predominantly insulating nature, we were able to create a protective layer with an adequate thickness that safeguarded the electrode while allowing the passage of the electric current at its tip.

Through comprehensive physical–chemical characterization, we observed a uniformly coated polymer with distinct functional groups that characterize PPPy/I. The physical–chemical properties and biocompatibility of PPPy/I make this electrode a highly innovative and technologically advanced development. This innovation is particularly evident in the improved long-term response observed in EGRs regarding spectral power and frequency compared to uncoated electrodes, and its biocompatibility could be useful in other applications, for example, coating invasive devices such as MEMS [40]. Tests on different types of coatings do not have favorable results; however, tests with conductive polymer coatings imply the need for non-toxic dopants, and, in general, their tissue–electrode interaction is something that can be improved [34]. This is an advantage of the coating with PPPy/I, since its good interaction with tissue has been proven [18].

Furthermore, we devised a methodology for creating the coating that ensures the long-term integrity of the electrode following implantation in the STN of the rat brain. Based on these findings, we anticipate that this innovative PPPy/I electrode coating technology could play a pivotal role in the development of more efficient and optimal electrodes for various therapies requiring invasive surgical procedures, particularly in patients with Parkinson’s disease and those undergoing deep brain stimulation treatments.

While our results show promise in achieving a uniform coating and enhancing the capture of electrical signals from the STN, it is imperative to delve deeper into the biological interactions. Future studies should encompass an evaluation of inflammatory processes during the electrode’s implantation period, the provision of electrical stimulation as therapy, and an assessment of tissue conditions after the designated study duration, which, in our case, spanned 10 weeks.

## Data Availability

The data presented in this study are available on request from the corresponding author.
